# TfAP-2 is required for night sleep in *Drosophila*

**DOI:** 10.1186/s12868-016-0306-3

**Published:** 2016-11-09

**Authors:** Mariya M. Kucherenko, Vinodh Ilangovan, Bettina Herzig, Halyna R. Shcherbata, Henrik Bringmann

**Affiliations:** 1 Max Planck Research Group Sleep and Waking, Max Planck Institute for Biophysical Chemistry, Am Fassberg 11, 37077 Göttingen, Germany; 2 Max Planck Research Group Gene Expression and Signaling, Max Planck Institute for Biophysical Chemistry, Am Fassberg 11, 37077 Göttingen, Germany; 3 Department of Genes and Behavior, Max Planck Institute for Biophysical Chemistry, Göttingen, Germany

**Keywords:** *Drosophila*, AP-2, Char syndrome, Insomnia, *C. elegans*

## Abstract

**Background:**

The AP-2 transcription factor APTF-1 is crucially required for developmentally controlled sleep behavior in *Caenorhabditis elegans* larvae. Its human ortholog, TFAP-2beta, causes Char disease and has also been linked to sleep disorders. These data suggest that AP-2 transcription factors may be highly conserved regulators of various types of sleep behavior. Here, we tested the idea that AP-2 controls adult sleep in *Drosophila*.

**Results:**

*Drosophila* has one AP-2 ortholog called TfAP-2, which is essential for viability. To investigate its potential role in sleep behavior and neural development, we specifically downregulated TfAP-2 in the nervous system. We found that neuronal TfAP-2 knockdown almost completely abolished night sleep but did not affect day sleep. TfAP-2 insufficiency affected nervous system development. Conditional TfAP-2 knockdown in the adult also produced a modest sleep phenotype, suggesting that TfAP-2 acts both in larval as well as in differentiated neurons.

**Conclusions:**

Thus, our results show that AP-2 transcription factors are highly conserved regulators of development and sleep.

**Electronic supplementary material:**

The online version of this article (doi:10.1186/s12868-016-0306-3) contains supplementary material, which is available to authorized users.

## Background

Sleep is an important part of human life and it is critical for learning performance and physical as well as mental well-being. Sleep disorders are widespread in modern societies but often the underlying mechanisms are not understood [1]. Sleep is controlled by the nervous system. Crucial to the induction of sleep in mammals are sleep-active sleep-promoting neurons such as those found in the Ventral Lateral Preoptic area (VLPO). These neurons express GABA and neuropeptides and are active preferentially at the onset of sleep to actively induce sleep [2]. However, little is known about the control of sleep at the molecular level in any system.

Experimental animal models are invaluable for analyzing medically relevant problems. In the recent past, invertebrate models have been established to study sleep. The use of invertebrate models has been made possible because careful analysis shows that sleep, as defined by behavioral criteria, could be found in the different phyla across the animal kingdom. These behavioral criteria include the absence of voluntary movement, an increased arousal threshold, and homeostatic regulation [3].

By applying these behavioral criteria, sleep was identified in the two major invertebrate model systems, *Drosophila melanogaster* and *Caenorhabditis elegans*. In *C. elegans*, quiescence behavior can be found during larval development as well as in adults [4]. The larval quiescence is called lethargus and is under developmental control and always precedes each of the four molts [5]. Lethargus quiescence also fulfills the behavioral criteria that define sleep, and thus this behavior has been proposed to be a sleep state [6, 7]. While initially the relationship between the various types of sleep was unclear, the ongoing molecular dissection of these processes suggests that they are carried out by similar molecular mechanisms [8]. This molecular data suggests that, despite the differences between the zoology of sleep in various systems, a common sleep-inducing mechanism evolved only once in evolution. This sleep machinery then radiated to give rise to the various types of sleep and different contexts in which sleep is employed. The common origin of sleep behavior enables studying the basic molecular mechanisms of sleep using simple animal models, which allows translating findings on sleep between different models.

In a brute-force screen for genes that control sleep behavior in *C. elegans*, the transcription factor Activator Protein 2 (AP-2) was found. Several AP-2 family genes have been found in vertebrates. The proteins encoded by these are very similar and can form heterodimers, which may contribute to their ability to regulate a wide variety of target genes. Deletion of APTF-1, one of four AP-2 paralogs in *C. elegans*, completely abolished locomotion quiescence. APTF-1 expressed in only few neurons including a single neuron that is called RIS. This neuron is sleep-active and sleep-promoting, and it expresses GABA and neuropeptides. APTF-1 is required for expression of inhibitory neuropeptides in RIS [9–11]. Thus, the RIS neuron in *C. elegans* appears to be similar to sleep-active neurons in mammals.

In humans, Char syndrome is found in patients that carry hemizygous loss-of-function mutations in TFAP-2beta, one of five AP-2 orthologs found in mammals [12, 13]. Whereas the loss of both alleles of TFAP-2beta is lethal, the loss of one of the alleles causes a haploinsufficiency phenotype that is characterized by abnormal limb, face, and heart development. These include a flat face with wide-set eyes, a patent ductus arteriosus, and a shortened or absent middle segment of the fifth finger. Sleep abnormalities in two families with Char syndrome have been reported, manifested either as sleepwalking or insomnia [14]. However, the sample size of the study was low. Also, the sleep phenotypes were not verified using sleep polysomnograms, making it difficult to understand the nature of the sleep problems in these patients. This is especially concerning as insomnia and sleepwalking are typically not linked. Maybe because the link between AP-2 and sleep was too weak, this initial observation was not followed in further publications.

The results from *C. elegans* on RIS support the view that sleep neurons are conserved regulators of sleep. If the role of AP-2 transcription factors in sleep is conserved, it will provide an entry point into studying sleep control in various systems. Also this would provide evidence for a common evolutionary origin of sleep neurons.

Here, we tested this idea directly by analyzing the role of AP-2 in sleep in *Drosophila*. In *Drosophila*, sleep has been mostly studied in adult flies. In this system, sleep behavior is under strong circadian control and is conveniently defined as an absence of movement that lasts more than 5 min. Flies show sleep behavior both during the middle of the day, called a siesta, and a more prominent sleep behavior during the night [15, 16].

*Drosophila* AP-2 displays a great degree of similarity with AP-2 proteins from other organisms. The DNA-binding domain is the most conserved part of the protein, and *Drosophila* AP-2 binds to the same DNA sequence as its mammalian counterparts [17]. Similarly to mouse AP-2 mutants and human patients with Char syndrome, *Drosophila AP*-*2* mutants are defective in joint development, where AP-2 acts in regulatory pathways that coordinate limb-growth with development of local and higher order aspects of limb-specific neural circuitry [18, 19]. Based on analyses of mouse, frog and chick AP-2 family members, vertebrate AP-2 transcription factors appear to play conserved roles in similar developmental contexts. The expression domains of AP-2 that seem most evidently conserved between fly and vertebrates are those in the nervous system, head and limbs. Considering conserved functions of vertebrate and invertebrate AP-2, we tested whether *Drosophila* AP-2 regulates sleep analogously to its *C. elegans* counterpart. We downregulated AP-2 in the *Drosophila* nervous system and found that AP-2 is specifically required for night sleep, and despite its role in development of the nervous system, it is also involved in the adult brain for sleep control.

## Methods

### Fly strains and genetics

RNA interference mutants *TfAP*-*2* ^*RNAi*^ (v41130 and v101552) were obtained from VDRC. Efficiency of *TfAP*-*2* downregulation was tested by RT-qPCR and the mutant *TfAP*-*2* ^*RNAi*^ (v101552), which had stronger *TfAP*-*2* downregulation (about 60%), was used in all the experiments. To downregulate TfAP-2 specifically in the nervous system and in subsets of neurons, the following driver lines (obtained from BDSC) were used: *elavGal4* (pan-neuronal driver)*, elavGal4;;UAS*-*Dcr*-*2* (drives expression of Dcr-2 in the nervous system)*, elavGal4;;tubGal80* ^*ts*^ (Gal80^ts^ restricts GAL4 expression when kept at 18 °C)*, c41Gal4* (expresses GAL4 in central brain and optic lobes), *TimGal4* (expresses GAL4 in the circadian rhythm pattern of the *timeless* gene)*, PDFGal4* (expresses GAL4 in PDF-expressing ventrolateral brain neurons), *201yGal4* (drives expression in γ and α/β mushroom body (MB) lobes), *cv*-*cGal4* (expresses GAL4 in cross veinless-c expressing neurons of fan shaped body involved in sleep regulation), *sNPFGal4* lines #49295, #48919, #49852 and #48880 (express GAL4 under control of sNPF regulatory sequences). *sNPFGal4* (#49852) has expression pattern similar to sNPF antibody staining, shows strong expression in the MBs and therefore was also used as MB-driver. To visualize the structure of all MB lobes, the *MB*-*247dsRed* line was used [20]. The *UAS*-*CD8GFP:UAS*-*nLacZ* line (a gift from Frank Hirth) was used to visualize *Gal4* expression. *w* ^*1118*^ flies, transgenic mutants crossed to *w* ^*1118*^ or driver lines crossed to *UAS*-*CD8GFP:UAS*-*nLacZ* were used as controls. To overexpress TfAP-2 we used *UAS*-*AP*-*2* transgenic flies (BDSC #23881) crossed to *Gal4*-expressing driver. Overexpression of TfAP-2 did not cause a sleep phenotype (Additional file 1: Table S2). To assay specificity of our newly generated anti-TfAP-2 antibodies we used an amorphic allele, *TfAP*-*2* ^*15*^ (BDSC #23721).

### *Drosophila* sleep monitors

A video monitor was used similar as described previously [21]. Briefly, flies were kept in DAM monitor tubes (Trikinetics) that contained ~1 cm of cornmeal sucrose medium on one side and this tube side was sealed with paraffin wax. After filling in a single male fly, the vial was closed with a foam plastic plug. 30 vials were taped onto a frosted glass plate and were placed inside an aquarium (50 cm × 60 cm × 50 cm) containing a 1 l beaker filled with distilled water as a moisture reservoir. For imaging we used a webcam (Zeiss), covered with an infrared filter (Delamax, 850 nm). An infrared lamp (Abus TV6855 LED) was placed half a meter behind the vials containing the flies to allow dia-illumination. A day-night cycle was displayed using an HL108S LED white-light floodlight controlled by a timer to provide a 12 h light–12 h dark cycle. The room was temperature-controlled to 25 °C. Fly activity was monitored and sleep parameters were extracted using Pysolo software. For experiments using infrared beam crossing, a standard protocol described by Rosato and Kyriacou [22] was used. Sleep parameters were analyzed using Microsoft Excel based counting macro [23].

### Immunohistochemistry

Brains were dissected in PBS and fixed in 4% formaldehyde (Polysciences, Inc.), adult and pupal for 30 min, larval for 15 min. Staining was performed as described [24]. The TfAP-2 antibody was commercially produced (Davids Biotechnolgie) as described [19]. Briefly, the c-terminal peptide of TfAP-2, CLDKSKIDNEKK, was synthetized, KLH conjugated, and antisera were generated in rabbits. The antibodies were then affinity-purified against the peptide and used in concentration 1:500 for Western blotting on embryonic tissue lysate (protein separation was done on 15% SDS-PAGE) or for immunostaining of the brain. In addition, the following antibodies were used: mouse anti-Fas II 1:20 (DSHB, marks γ and α/β MB lobes), mouse anti-PDF 1:20, rabbit anti-sNPF 1:1000 (a gift from Jan-Andrianus Veenstra), and chicken anti-GFP 1:1000 (Abcam). Alexa 488, 568, or 633 goat anti-mouse, anti-rabbit, anti-chicken (1:500, Molecular Probes). DAPI was used to visualize nuclei.

### Imaging and image analysis

Images were obtained with a confocal laser-scanning microscope Zeiss LSM700 and processed with Adobe Photoshop. Mushroom body (MB) size was measured from maximum intensity projection images for at least 10 FasII stained MB lobes taken at the same magnification. By using ZEN 2011 software, FasII positive MB lobes were outlined and their area in µm^2^ was calculated. Data were presented as relative to control. The statistical significance of the observed difference was calculated using a two-tailed Student’s *t* test. The intensity of TfAP-2 antibody staining in large ventral lateral neurons (l-LNv) was quantified by ZEN 2011 software from single section images taken across anti-PDF-marked cells. To calculate absolute intensity of TfAP-2 expression specifically in l-LNv neurons, the background staining (measured from the same size area in the neighboring non-PDF-expressing cells) was subtracted. To test whether AP-2 expression was downregulated in l-LNv neurons by expressing *TfAP*-*2 RNAi* with *TimGal4* driver, we compared TfAP-2 antibody staining intensity of *TimGal4* > *TfAP*-*2* l-LNv neurons with control l-LNv neurons (*TimGal4* > *GFP*). Data were collected for at least 10 l-LNv neuronal clusters. The significance of AP-2 down regulation was calculated using one-tailed Student’s *t* test.

### RNA preparation and real time quantitative PCR

To determine the efficiency of TfAP-2 downregulation in *TfAP*-*2* ^*RNAi*^ mutants and the effect of TfAP-2 on other gene expression, mRNA levels quantitative reverse transcription PCRs (RTqPCR) were performed on total RNA derived from whole *Drosophila* bodies. RNA was extracted from flies using the TRIzol reagent (Invitrogen), followed by reverse transcription using the High Capacity cDNA Reverse Transcription kit (Applied Biosystems) following the manufacturer’s protocol. mRNA levels were tested with *RpL32* or *Act5c* as an endogenous controls for the qPCR using Fast SYBR Green master mix on a Step One Plus 96 well system (Applied Systems). Following primers were applied: *RpL32* forward AAGATGACCATCCGCCCAGC; *RpL32* reverse GTCGATACCCTTGGGCTTGC; *Act5c* forward GTGCACCGCAAGTGCTTCTAA; *Act5c* reverse TGCTGCACTCCAAACTTCCAC; *TfAP*-*2* forward ATAGCCGAAGTACAGCGTCG; *TfAP*-*2* reverse CCAGCTTCTCCCTCAACAGG; *sNPF* forward: CACACCATCTTCGAGCTGAATAA; *sNPF* reverse TTTTCAAACATTTCCATCGT. All reactions were run in triplicate. The threshold cycle (C_T_) is defined as the fractional cycle number at which the fluorescence passes the fixed threshold. The ΔC_T_ value was determined by subtracting the average *RpL32* or *Act5c* C_T_ values from the *AP*-*2* and *sNPF* C_T_ values. The ΔΔC_T_ value was calculated by subtracting the ΔCT of the control sample from the ΔC_T_ of the suspect sample (*TfAP*-*2* ^*RNAi*^ mutant). The relative amount of mRNA was then determined using the expression $2^{{ - {\Delta \Delta }C_{\text{t}} }}$. Significant changes in mRNA levels were calculated using two-tailed Student’s *t* test.

### Phylogenetic tree generation

The multiple alignment and phylogenetic tree were computed with Clustal W phylogeny by pasting in the following sequences [25]: *tfap*-*2alpha*(*Homo sapiens*) accession EAW55251, *tfap*-*2epsilon*(*H. sapiens*) accession NP_848643, *tfap*-*2beta*(*H. sapiens*) accession CAA64990, *tfap*-*2delta*(*H. sapiens*) accession NP_758438, *tfap*-*2gamma*(*H. sapiens*) accession NP_003213, *aptf*-*1*(*C. elegans*) accession NP_495300, *aptf*-*2*(*C. elegans*) accession NP_001255740, *aptf*-*3*(*C. elegans*) accession NP_495819, *aptf*-*4*(*C. elegans*) accession NP_495818, *tfap*-*2*(*Nematostella vectensis*) accession XP_001633944, *tfap*-*2*(*Trichoplax adhaerens*) accession XP_002114137, *tfap*-*2*(*D. melanogaster*) accession NP_649336.

## Results

### *TfAP*-*2* is required for night sleep

**Fig. 1:**
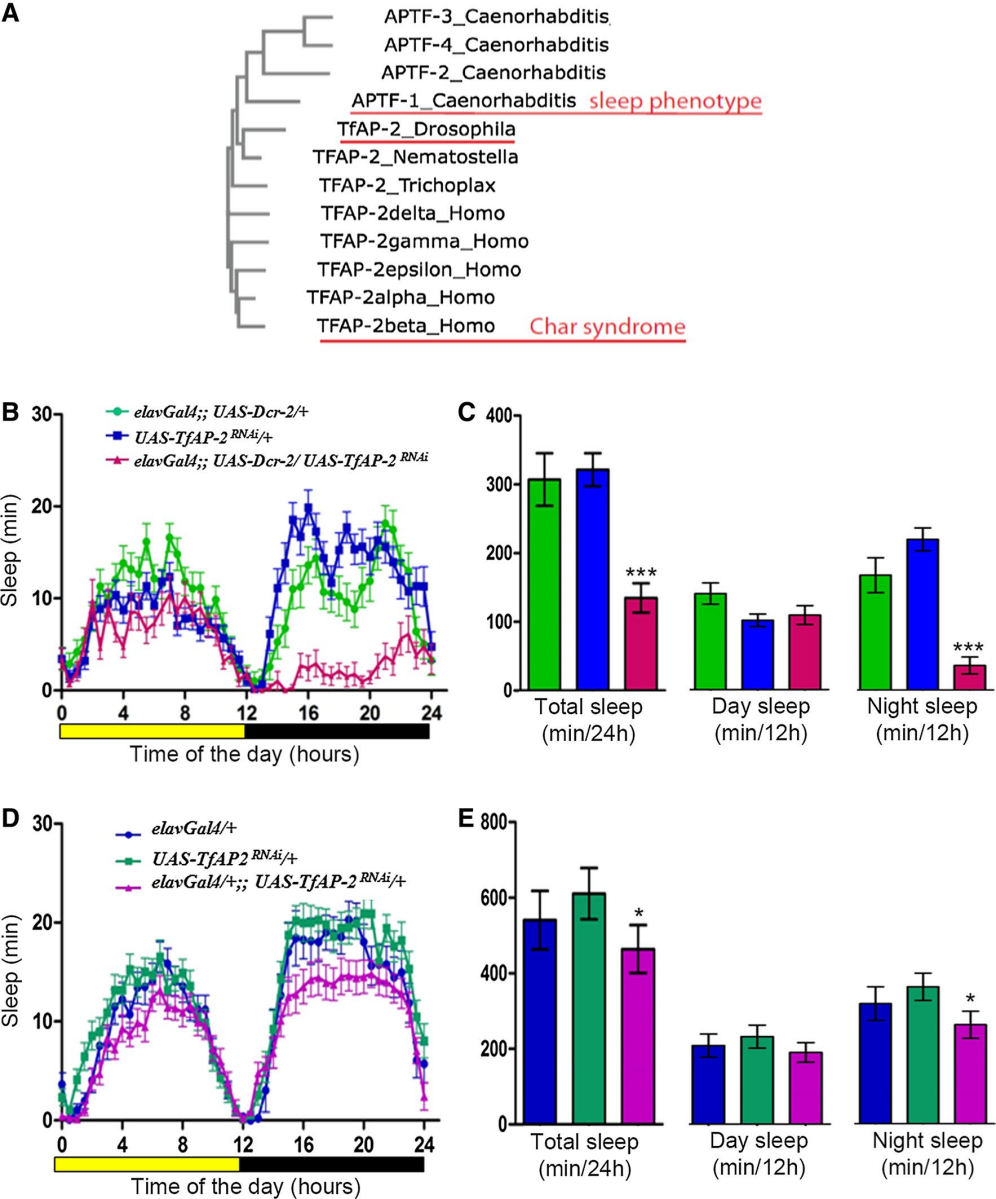
Neuronal TfAP-2 is required for night sleep. **A** TfAP-2 is a well-conserved transcription factor present in most multicellular organisms of the animal kingdom. The multiple alignment and phylogenetic tree were computed with Clustal W phylogeny [25]. **B** The sleep profile of adult flies was generated using a video monitoring protocol and extracted by PySolo. Expression of Dcr-2 and *TfAP*-*2* ^*RNAi*^ were induced in neurons throughout the life history of these flies. **C** Quantification of sleep in Dcr-2 and *TfAP*-*2* ^*RNAi*^ flied. **D** Sleep profile of flies was recorded using Trikinetics infra-red beam crossing and processed by a counting macro. Neuronal knockdown of TfAP-2 results in drastic reduction of night sleep in the presence of Dcr-2. The phenotype is weaker when knockdown is achieved without exogenous Dcr-2. *UAS*-*TfAP*-*2* ^*RNAi*^ (v101552) strain was used for knockdown of TfAP-2 in these experiments. **E** Quantification of sleep parameters measured in **d**. ***p < 0.0001 Student *t* test. *p < 0.05 determined by one way ANOVA, Dunnet post hoc test

AP-2 transcription factors are highly conserved among metazoans. The *Drosophila* genome contains only one *AP*-*2* gene that is called *TfAP*-*2* (Fig. 1A) [12]. Ubiquitous early knockdown of this gene is lethal at the larval or pupal stage, and hypomorphic loss-of-function mutation affects limb and brain development [19]. To test for sleep phenotypes, we generated viable mutants using RNAi to knock down *TfAP*-*2* specifically in the nervous system [26, 27]. We followed the sleep behavior of the flies using established sleep monitor systems. For the RNAi experiments, we used transgenic flies that expressed a double-stranded RNAi hairpin construct that targets *TfAP*-*2* under the control of the upstream activating sequence (*UAS*). To drive expression in the entire nervous system, we used the *elavGal4* driver. For an initial experiment we also included *Dcr*-*2* expression, which can increase the efficiency of RNAi [28]. We used two independent RNAi constructs, *TfAP*-*2* ^*RNAi(v41130)*^ and *TfAP*-*2* ^*RNAi(v101552)*^ [29]. To monitor sleep, we used a video-based system and analyzed the behavior of male flies during a 12 h light–12 h dark cycle [21]. As controls, we used the parental strains. Control flies showed some sleep during the middle of the day, and substantial sleep during the night (Fig. 1B). However, *TfAP*-*2* ^*RNAi*^ reduced night sleep specifically. Whereas there was a modest reduction for *TfAP*-*2* ^*RNAi(v41130)*^, night sleep was almost completely abolished in *TfAP*-*2* ^*RNAi(v101552)*^ flies, with some individuals completely lacking any detectable night sleep (Fig. 1B, C; Additional file 1: Figure S1). To measure the knockout strength of the RNAi, we performed quantitative real-time PCR for *TfAP*-*2* mRNA on *TfAP*-*2* ^*RNAi*^ flies. mRNA levels for *TfAP*-*2* were reduced by 22% in *TfAP*-*2* ^*RNAi(v41130)*^ and by 58% in *TfAP*-*2* ^*RNAi(v101552)*^ (Additional file 1: Figure S2, Table S1). Even though *Dcr*-*2* has been found to be useful for increasing the RNAi, it can also increase off target effects [29]. Thus, we repeated the sleep measurements without *Dcr*-*2* over-expression and did not use *Dcr*-*2* for any further experiments. We also switched to a sleep monitor system that detects locomotion based on infrared-beam arrays, which was easier to use in comparison to the video monitor [15, 16, 30]. This experiment reproduced the sleep phenotype for neural *TfAP*-*2* ^*RNAi*^. However, the observed phenotypes appeared slightly weaker when compared with *Dcr*-*2* expression (Fig. 1D, E). Thus, TfAP-2 is specifically required for night sleep, but not for siesta sleep. The sleep phenotype strength correlated with the knockout strength for the two different RNAi constructs, suggesting that the effects are indeed caused by *TfAP*-*2* knockdown. Like *C. elegans aptf*-*1*, *Drosophila TfAP*-*2* acts in the nervous system to control sleep.

### *TfAP*-*2* is expressed in the nervous system

**Fig. 2:**
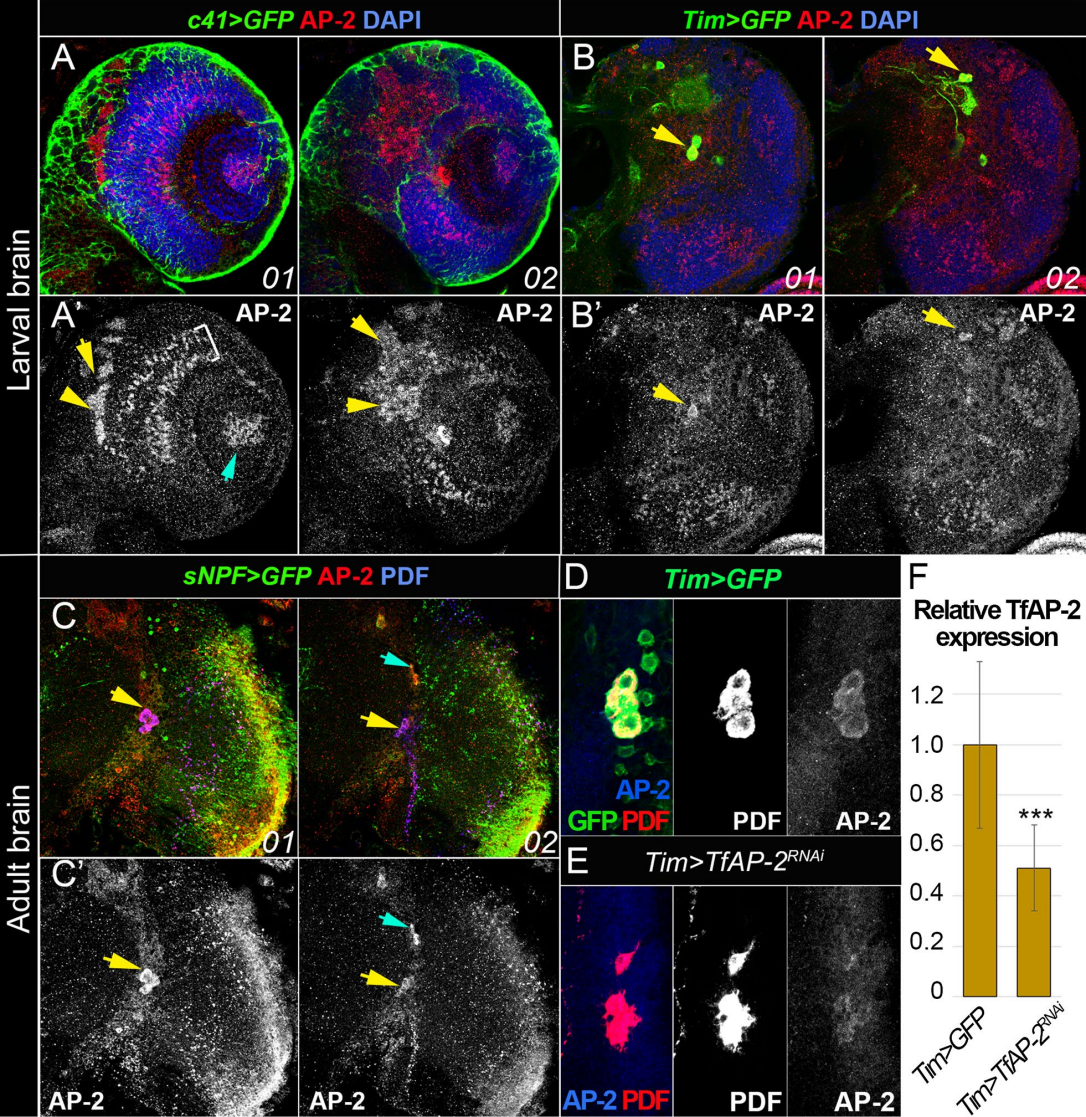
*TfAP*-*2* is expressed in larval and adult *Drosophila* brain. Antibodies against TfAP-2 were used to reveal the TfAP-2 expression pattern in developing larval (**A**, **B**) and adult (**C**) *Drosophila* brains. **A** *c41Gal4* driver expressing membrane bound GFP was used to mark contour and the central brain regions. In 3rd instar larvae TfAP-2 was found to have strong expression in the subsets of neurons located in the central brain (*yellow arrows*), medulla (*parenthesis*) and lamina (*blue arrows*) regions. **B** In larval brain TfAP-2 is also expressed in some of *TimGal4* > *CD8GFP* expressing neurons (*arrows*). **C** In adult brain TfAP-2 protein can be found in ventral lateral neurons marked with *sNPF* > *CD8GFP* (*blue arrows*) and anti-PDF staining (*yellow arrows*). **D** TfAP-2 expression in l-LNv neurons marked with *TimGal4* > *CD8GFP* and anti-PDF in adult brain. **E** TfAP-2 expression in l-LNv neurons marked with anti-PDF in *TimGal4* > *TfAP*-*2RNAi* mutant adult brain. **F** *Bar graph* shows relative intensity of TfAP-2 antibody staining in PDF-expressing neurons for *TimGal4* > *CD8GFP* (control) and *TimGal4* > *TfAP*-*2RNAi* animals. Antibody staining intensity (Mean ± Average deviation) is 1 ± 0.33 (n = 27) for control, and 0.51 ± 0.17 (n = 10) for *TfAP*-*2RNAi.* n, number of l-LNv neuronal clusters. Statistical significance of TfAP-2 downregulation was calculated by Student *t* test, p = 0.00023

Because *TfAP*-*2* was required in the nervous system to control sleep, we wanted to know more about the expression of this transcription factor. We raised an antibody against *TfAP*-*2* and investigated the expression pattern using immunostaining in both the larval and adult nervous system. As published previously, we observed a strong AP-2 staining in the leg discs implying specificity of the newly generated antibody (Additional file 1: Figure S3 A, B). To further test the specificity of newly generated antibody we stained individuals carrying the amorphic allele *TfAP*-*2* ^*15*^ and observed that in comparison to control (*TfAP*-*2* ^*15*^ */TM6,Tb*), *TfAP*-*2* ^*15*^ homozygous mutant brains did not express TfAP-2 (Additional file 1: Figure S3 C, D). In the 3rd instar larvae, we found strong expression in a subset of neurons in the central brain region, but also in the medulla and lamina (Fig. 2A). The expression partly overlapped with Tim-expressing neurons (Fig. 2B). In adult brains, the expression levels of AP-2 appeared much lower compared with larval brains. Importantly, we found TfAP-2 protein to be expressed in sleep-related sNPF-, Tim- and PDF-expressing neurons (Fig. 2C, D) and by driving *TfAP*-*2RNAi* with *TimGal4* driver we were able to significantly reduce TfAP-2 expression specifically in l-LNv neurons measured by the intensity of TfAP-2 antibody staining (Fig. 2D–F) and RTqPCR (Additional file 1: Table S1). Thus, TfAP-2 is expressed in larval and adult neurons and the intensity of AP-2 expression is decreased during adulthood in comparison to pre-adult stages.

### *TfAP*-*2* is required for nervous system development

**Fig. 3:**
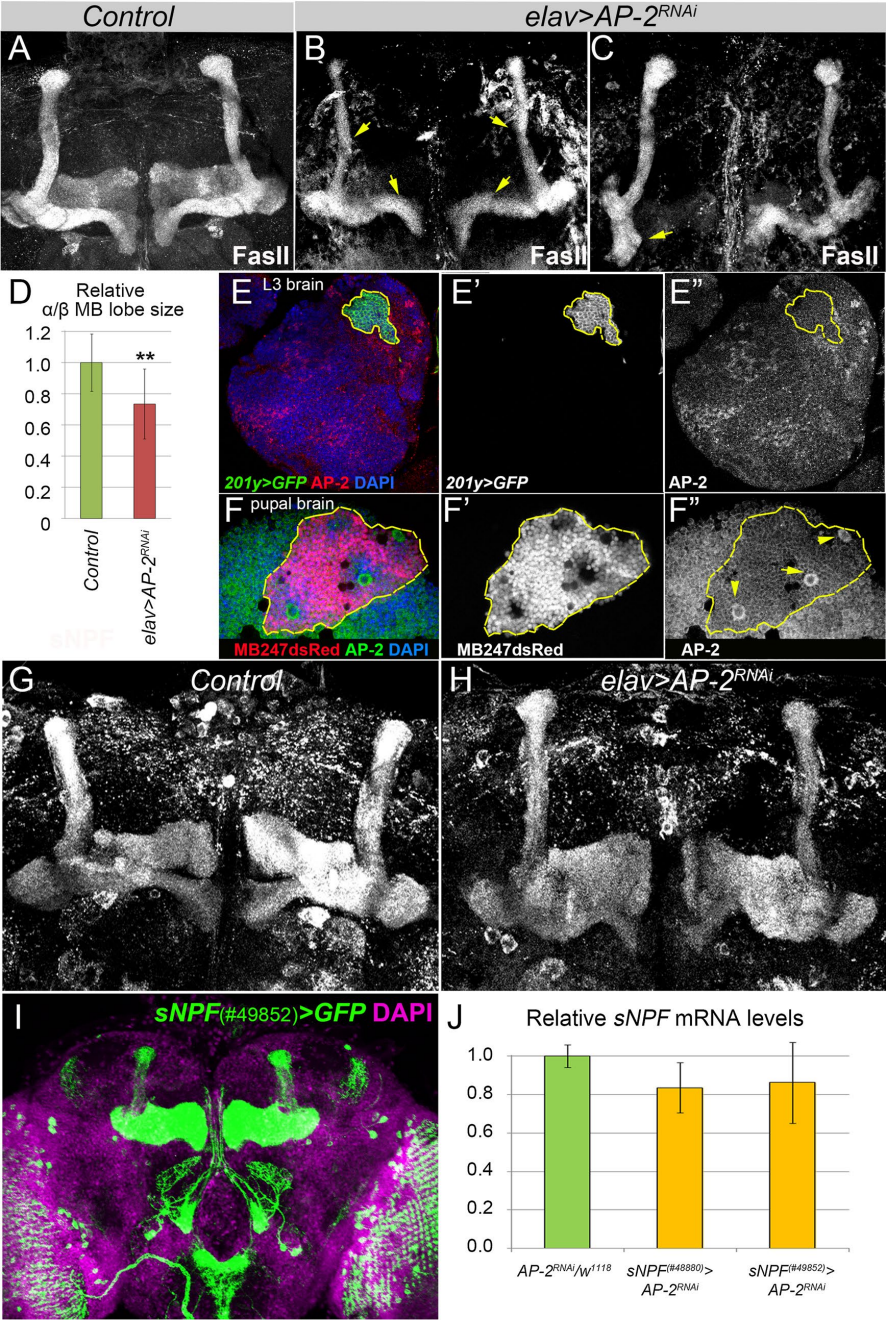
*TfAP*-*2* downregulation affects brain development. Downregulation of *Drosophila TfAP*-*2* with pan-neuronal driver *elavGal4* caused significant mushroom body developmental defects. In comparison to *Control* (**A**) *TfAP*-*2* mutant brains (*elav* > *AP*-*2* ^*RNAi*^ *v101552*) had slimmer (**B**, *arrows*) and/or disrupted (**C**, *arrows*) axonal projections of MB neurons visualized by anti-FasII staining (a marker for γ and α/β MB lobes). **D** The *bar graph* shows relative size of α/β MB lobes. Analysis of TfAP-2 protein expression in larval (**E**) and pupal (**F**) developing brains revealed no significant TfAP-2 expression in MB neuronal cell bodies (**E**–**E**″ marked with γ lobe marker *201Gal4* > *CD8GFP*) and (**F**–**F**″ marked with γ, α‘/β‘and α/β MB lobes marker *MB247dsRed*) but considerable TfAP-2 expression in pupal MB neuroblasts (**F**, *arrows*). MB calyces are outlined. (**G**, **H**) *TfAP*-*2* reduction with *elavGal4* driver did not have prominent effect on sNPF protein localization in the MBs and sNPF neuron-specific downregulation of *TfAP*-*2* (**I**, **J**) did not cause significant reduction in sNPF mRNA levels

The strong expression of *TfAP*-*2* in larvae suggested that this transcription factor plays a role in the development of the nervous system. Thus, we investigated whether neuron-specific downregulation of *TfAP*-*2* affects brain development, which would result in the appearance of abnormal structures in the adult brain. We focused especially on the adult mushroom body (MB) and sNPF-expressing neurons, as these compartments have been implicated in sleep control [31–33]. We found that the mushroom body morphology was severely affected. Axonal projections of α/β MB lobes marked by FasII expression were slimmer or disrupted, which affected the average volume of MB lobes (Fig. 3A–D). Following this observation we marked MB in larval (with *201y Gal4* > *GFP*) and pupal (with *MB247dsRed*) brains and analyzed TfAP-2 expression pattern. TfAP-2 protein was expressed in mushroom body neuroblasts during the pupal stage (the time point of α/β MB lobes formation), but not in larvae (Fig. 3E, F). Thus, consistent with previous findings [19], knockdown of *TfAP*-*2* influenced MB neural morphogenesis. Since MB neurons express plenty of short neuropeptide F (sNPF, Fig. 3G), which has been shown to regulate sleep in *Drosophila* [33], we evaluated sNPF expression by detecting its protein and mRNA levels in control and *TfAP*-*2* mutant (Fig. 3G–J; Additional file 1: Table S1). We did not observe significant effect of *TfAP*-*2* downregulation on sNPF expression suggesting that TfAP-2 controls sleep independently of sNPF. Based on the TfAP-2 expression pattern, we hypothesized that TfAP-2 acts in either MB-, sNPF-, Tim- or PDF-expressing neurons. We thus used these tissue-specific *Gal4* lines as well as *cv*-*cGal4* driver, which is expressed in known sleep-controlling *crossveinless*-*c* neurons [34] to downregulate *TfAP*-*2* and to monitor sleep behavior. As GABAergic neurons play a role in sleep control, we also tested the *gad*-*Gal4* driver [35]. A recent study reported that TfAP-2 regulates *Drosophila* aggression behavior by acting in octopaminergic neurons [26, 27, 36]. In addition, it has been previously shown that octopamine regulates sleep in *Drosophila* and we hence also tested *tdc*-*Gal4* [37]. None of these experiments gave a sleep phenotype, suggesting that either the knockdown strength was not strong enough or that *TfAP*-*2* is required in other neurons that are yet to be identified or acts redundantly in several neuron types (Additional file 1: Table S2).

### TfAP-2 is required for sleep also in the adult brain

**Fig. 4:**
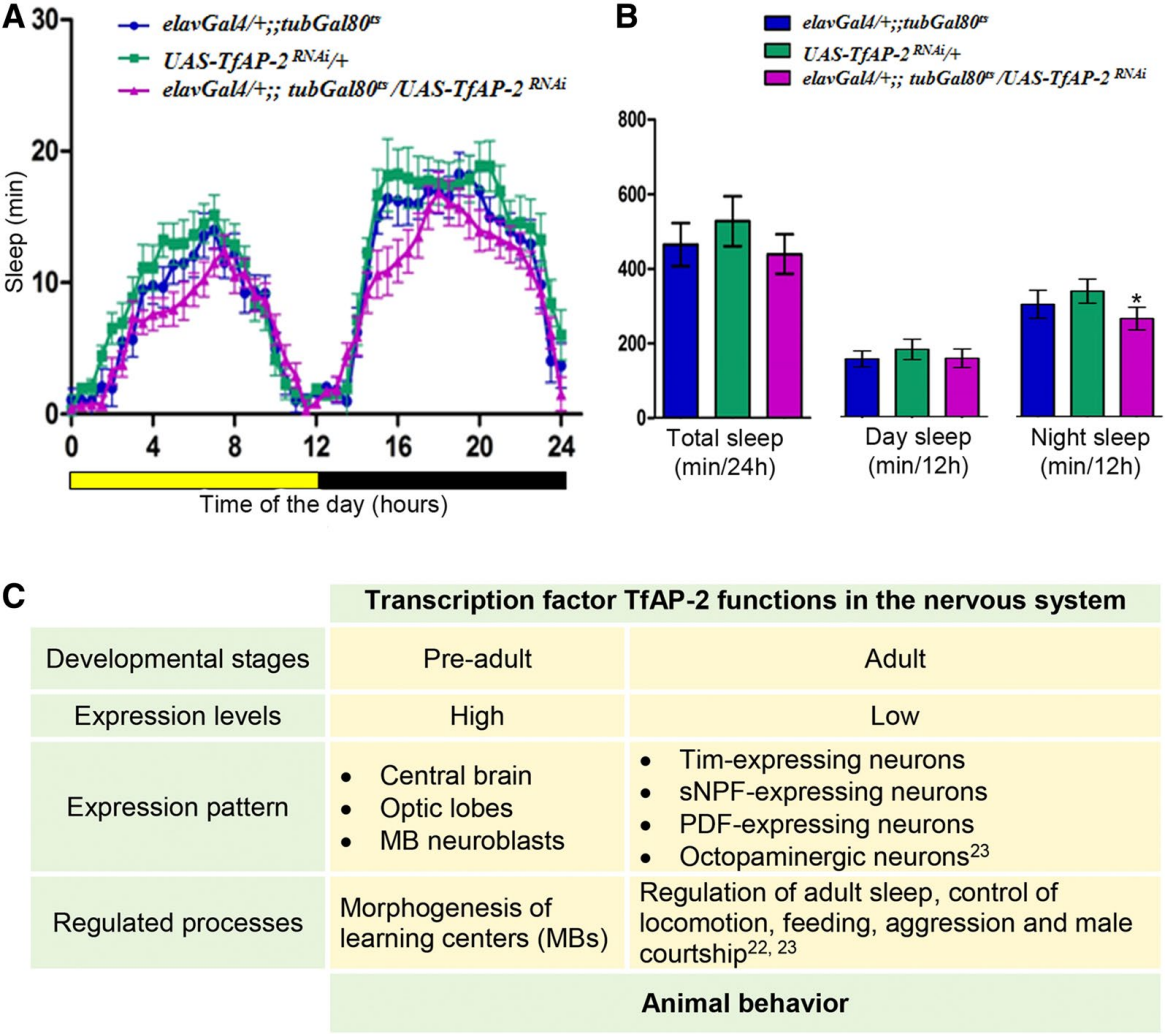
TfAP-2 is required in the adult brain for effective night sleep. **A** Pan-neuronal knockdown of *TfAP*-*2* in adult neurons reduces night sleep. In order to restrict knockdown of *TfAP*-*2* in neurons only during adulthood a temperature-sensitive Gal80 allele was used. These flies were reared at permissive temperature of 18 °C and then switched to restrictive temperature of 30 °C after adult emergence. The sleep assay was also conducted at 30 °C. *UAS*-*TfAP*-*2* ^*RNAi*^ (v101552) strain was used for knockdown of TfAP-2 in these experiments. **B** Total sleep, relative day sleep and relative night sleep of the parental controls and mutant flies. **C** TfAP-2 functions in larval and adult *Drosophila* brain. *p < 0.05 determined by one way ANOVA, Dunnet post hoc test

While AP-2 appears to have a role in development, it may exert its function in sleep not only through a role in development, but may rather work in differentiated neurons. To test this idea, we knocked down *TfAP*-*2* specifically in adults and tested for sleep phenotypes. For this experiment, we used a temperature-sensitive suppressor of *Gal4*, *Gal80* ^*ts*^, which was expressed ubiquitously. Under normal growth conditions *Gal80* ^*ts*^ represses the expression of the RNAi construct but can be inactivated by increasing the temperature to 30 °C. We grew larvae under permissive temperatures and then knocked down *TfAP*-*2* specifically in adults by shifting the adults to the higher temperature. We found that adult-specific neural *TfAP*-*2* ^*RNAi*^ also caused a reduction in night sleep. However, the reduction of sleep was much smaller compared with permanently active neural *TfAP*-*2* downregulation (Fig. 4A, B). This suggests that *TfAP*-*2* is required during neural development but also plays a role in differentiated neurons of the adult. Because *TfAP*-*2* expression in adults is very low, the neurons through which *TfAP*-*2* acts in adults to control sleep are difficult to identify with available immunohistochemistry approaches. Thus, TfAP-2 is required in neurons that remain to be identified.

## Discussion

Both *C. elegans* and *Drosophila* are well-established powerhouses of molecular biology, and sleep research in these systems is highly attractive. Both *Drosophila* and *C. elegans* belong to the group of molting animals (Ecdysozoa) [38]. The nervous system of *Drosophila* is more complex and contains approximately 100.000 neurons compared with 302 neurons and a mapped connectivity in *C. elegans* [39]. Thus, while *C. elegans* is easier to use for circuit analysis, *Drosophila* can be used to study more complex aspects of brain regulation. In *Drosophila*, sleep behavior is light dependent and coupled to a clear day-night cycle. In *C. elegans*, genes homologous to the genes that control circadian behavior in other organisms also control sleep behavior. However, the periodicity in *C. elegans* is shorter and the rhythm is not entrained by light [40, 41]. Whereas the *Drosophila* sleep system can be used to study the control by the day-night cycle, the developmental sleep system of *C. elegans* facilitates genetic analyses. Finally, the transparency of *C. elegans* also allows non-invasive functional brain imaging [42]. Thus, both *C. elegans* and *Drosophila* have their unique strengths, which nicely complement each other. These models have the capacity to ultimately solve molecular mechanisms underlying sleep control, and because of the common evolutionary origin of sleep, these findings may be translatable to the mammalian system and thus may help understand the causes of human sleep disorders.

In *Drosophila,* transcription factor TfAP-2 is not only a potent regulator of developmental processes, but it is also expressed in the adult brain and controls multiple behavioral processes (Fig. 4C). Here we show that TfAP-2 is required in neurons for adult sleep in *Drosophila* (Fig. 4C). Thus, AP-2 transcription factors are conserved regulators of sleep. Generally, transcription factors have been crucial in resolving evolutionary relationships. A good example is PAX6, which is required for eye development in various species. This led to the view that all eyes, despite their differences in different species, have evolved from an ancestral prototypic eye [43]. Similarly, the conserved role of AP-2 in sleep control provides strong support for the view that larval sleep behavior in *C. elegans* and adult night sleep in *Drosophila* share a common evolutionary origin. Besides a strong role of TfAP-2 in development, this transcription factor appears to also play a role in sleep in differentiated adult neurons. Unfortunately, the weak expression in the adult brain compared with the strong expression during development has impaired a straightforward identification of the neurons in which TfAP-2 acts in sleep control. More sensitive localization analyses combined with cell-specific RNAi may solve this problem in the future.

## Conclusions

Sleep is found in all animals that have a nervous system but little is known about the evolutionary origins of sleep. The transcription factor AP2 is linked to Char disease and insomnia in humans, and is required for sleep neuron function in *C. elegans*. Here we show that AP2 is crucially required for night sleep in *Drosophila*. Despite regulating larval development, AP2 is also required in the adult brain for sleep control. Thus, our work shows that AP2 transcription factors are conserved regulators of sleep and this work thus provides strong support for a common evolutionary origin of sleep-controlling mechanism. We establish a system in which sleeping disorders associated with Char disease can be studied in a highly accessible system. Because *Drosophila* and *C. elegans* are established model systems for the molecular dissection of biological processes, it should be possible to solve the molecular role of AP-2 in these systems. Ultimately, further understanding how AP-2 works in sleep control may shed light on sleep disorders such as those found in Char syndrome.

## Additional file

**Additional file 1.** Supplementary information.

## Abbreviations

VLPO: ventral lateral preoptic area
AP-2: activator protein 2
