# Supplementary material for: TfAP-2 is required for night sleep in Drosophila
Source: BMC Neurosci. 2016 Nov 9;17:72. doi: 10.1186/s12868-016-0306-3 (PMC5103423; doi:10.1186/s12868-016-0306-3)
Supplement: Supplementary file 1 — Additional file 1. Supplementary information. [file 12868_2016_306_MOESM1_ESM.docx]

**Supplementary Figures**

**Supplementary Figure S1**

**
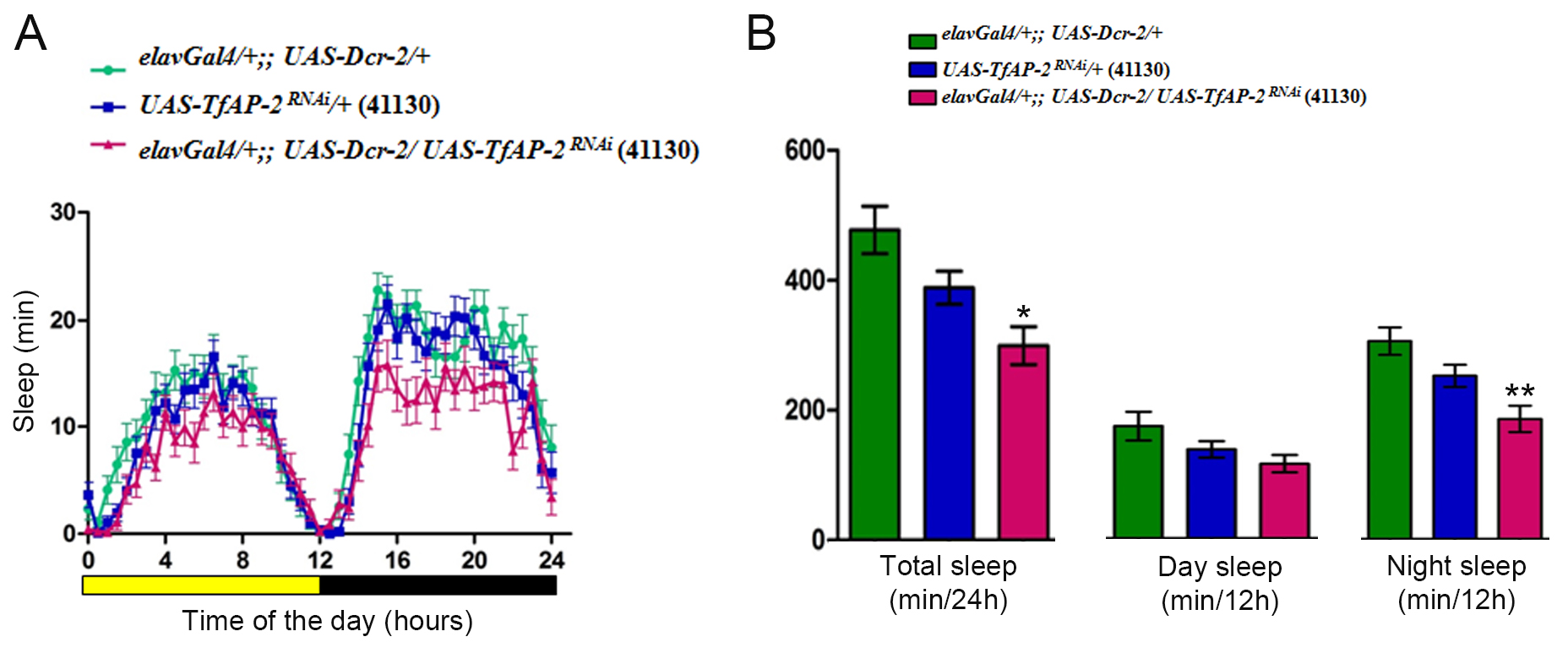
**

**Figure S1. *TfAP-2^RNAi(v41130)^* causes reduced night sleep**

*UAS-TfAP-2 ^RNAi^* (v41130) strain shows a weaker reduction of night sleep. The strength of the UAS responder strain affects the sleep phenotype very weakly due to the less efficiency of knockdown (see also Supplemental Figure S2 and Supplemental Table 1).

**Supplementary Figure S2**

**
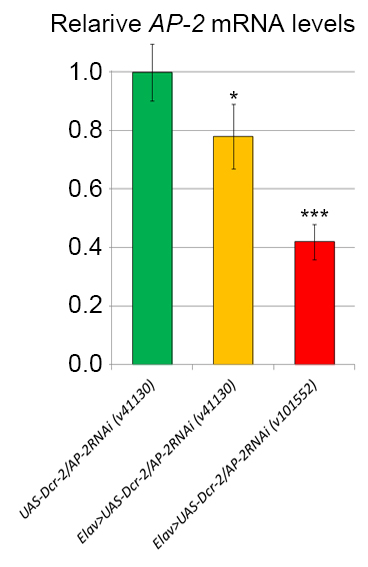
**

**Figure S2. *TfAP-2* mRNA levels are downregulated in *AP-2^RNAi^* mutants**

The efficiency of *TfAP-2* downregulation by RNA interference tested with RT-qPCR is represented in the bar graph. Two RNA interference encoding transgenes targeting *TfAP-2* in different loci were expressed specifically in the nervous system using pan-neuronal *elavGal4* driver. *elav>UAS-Dcr2/AP-2^RNAi^ (v41130)* reduces *TfAP-2* expression levels by 22 % and *elav>UAS-Dcr-2/AP-2^RNAi^ (v101552)* by 58 % when compared to appropriate control *elav>UAS-Dcr2.*

**Supplementary Figure S3**

**
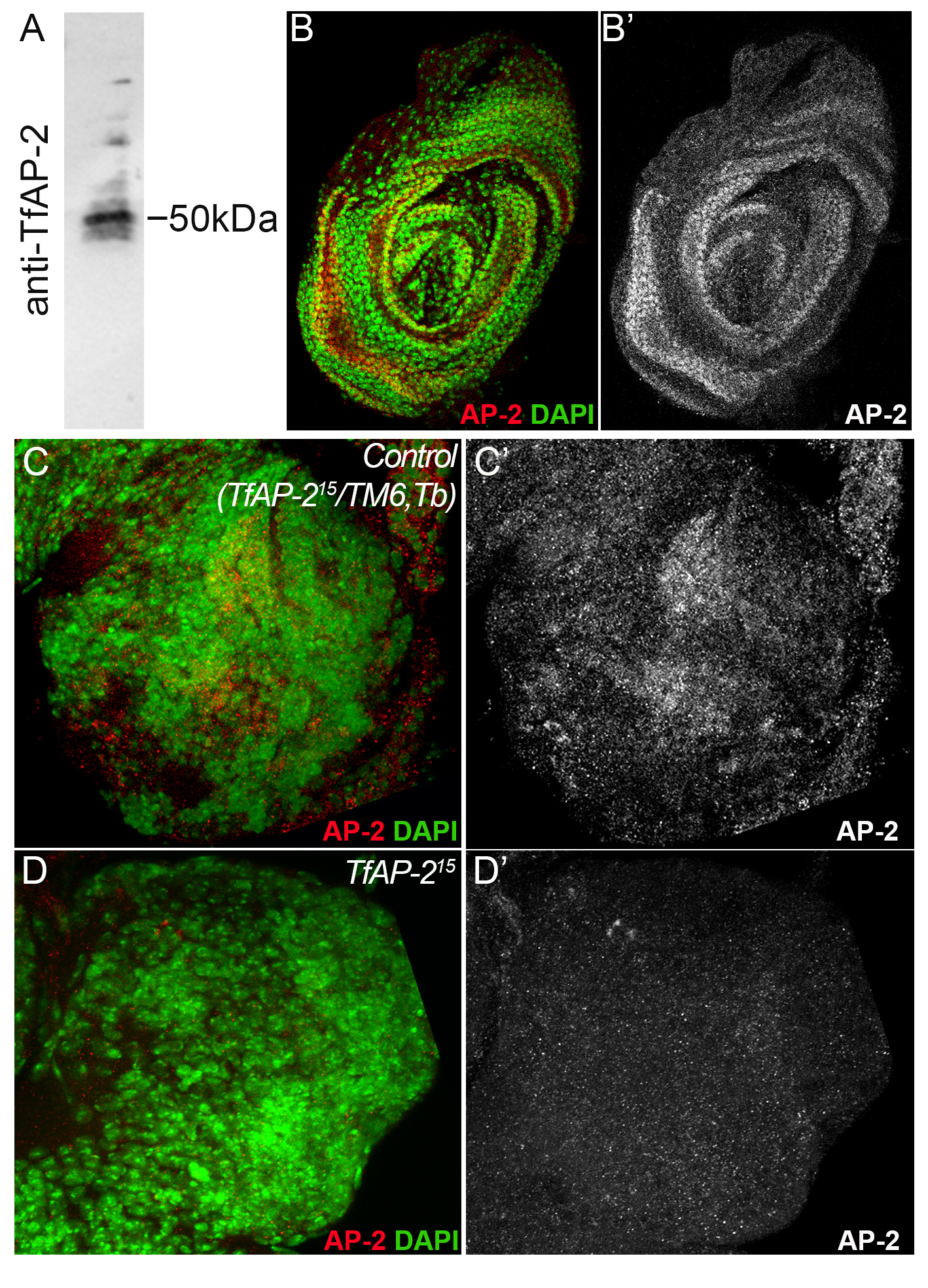
**

**Figure S3. Verification of newly generated anti-TfAP-2 antibodies**

The efficiency of newly raised antibodies against TfAP-2 was tested on Western blot from *Drosophila* embryo extract (A). The band of approximately 50 kDa size corresponding to the size of *Drosophila* TfAP-2 protein is detected. (B) Immunostaining of larval tissues showed similar to previously reported[[44](#_ENREF_44)] TfAP-2 expression in the leg disc. (C) Second instar larval brain of control (*TfAP-2^15^/TM6,Tb*) animal shows TfAP-2 expressing areas in the central brain. (D) Second instar larval brain of *TfAP-2^15^* homozygous mutant do not express TfAP-2. Note, the second instar larval developmental stage was chosen for analysis due to lethality of *TfAP-2^15^* animals at later stage.

**Supplemental Table S1. Expression levels of *AP-2, Tim* and *sNPF* in *AP-2* mutants**

|  | **Genotype** | **Target** | **ΔC_T_**  **(Mean ± SD)** | **ΔΔC_T_**  **(Mean ± SD)** | **Relative mRNA levels**  **(Mean ± SD)** | **Statistics**  **p-Value** |
| --- | --- | --- | --- | --- | --- | --- |
| **Experiment 1** | *UAS-Dcr-2/AP-2^RNAi^*  (Control),  males, whole body | *AP-2* | 9.90±0.13 | 0.01±0.14 | 0.99±0.09 | - |
|  | *elav>UAS-Dcr2/*  *AP-2^RNAi^ (v41130),*  males, whole body | *AP-2* | 10.26±0.21 | 0.36±0.21 | 0.78±0.11 | 0.03* |
|  | *elav>UAS-Dcr-2/*  *AP-2^RNAi^ (v101552)*  males, whole body | *AP-2* | 10.54±0.53 | 1.26±0.23 | 0.42±0.07 | 5.62x10^-4^  *** |
| **Experiment 2** | *Tim>GFP*  (Control),  females, whole body | *AP-2* | 7.11±0.13 | 0.00±0.12 | 1.00±0.09 | - |
|  | *Tim>*  *AP-2^RNAi^ (v101552),*  females, whole body | *AP-2* | 8.13±0.01 | 1.02±0.02 | 0.49±0.01 | 6.4x10^-4^  *** |
| **Experiment 3** | *AP-2^RNAi^/w^1118^*  (Control),  males, whole body | *sNPF* | 5.58±0.06 | 0.00±0.06 | 1.00±0.04 | - |
|  | *sNPF(48880)>*  *AP-2^RNAi^ (v101552)*  males, whole body | *sNPF* | 5.84±0.13 | 0.26±0.13 | 0.84±0.08 | 0.08 |
|  | *sNPF(49852)>*  *AP-2^RNAi^ (v101552)*  males, whole body | *sNPF* | 5.79±0.21 | 0.21±0.22 | 0.87±0.13 | 0.27 |

**Supplemental Table S1. Expression levels of *AP-2, Tim* and *sNPF* in *AP-2* mutants** *RpL32* was used as endogenous controls in the Experiment1 and *Act5c* in Experiments 2 and 3. Statistics was calculated from the triplicates using two-tailed Student’s t-test, *p<0.05; ***p<0.00

**Supplemental Table S2. Sleep phenotype quantification**

| **Genotype** | | **Number of analyzed animals (n)** | **Total sleep, min**  **(AVE±SEM)** |
| --- | --- | --- | --- |
| 1 | *UAS-TfAP2^RNAi^(v101552)/+* | 21 | 610.45±68.05 |
|  | *elav Gal4/+* | 20 | 540.35±77.21 |
|  | *elav Gal4/+;;UAS-TfAP2^RNAi^(v101552)/+* | 27 | 464.09±63.14 * |
|  | *UAS-TfAP2^RNAi^ (v101552)/+* | 97 | 568.72±39.23 |
|  | *Tim Gal4/+* | 16 | 541.20±53.44 |
|  | *Tim Gal4/+; UAS-TfAP2^RNAi^(v101552)/+* | 21 | 537.34±42.37 |
|  | *PDF Gal4/+* | 29 | 602.04±40.81 |
|  | *PDF Gal4/+; UAS-TfAP2^RNAi^(v101552)/+* | 32 | 590.74±37.66 |
|  | *GAD1 Gal4/+* | 18 | 469.08±61.45 |
|  | *GAD1 Gal4/+ ;UAS-TfAP2^RNAi^(v101552)/+* | 24 | 512.26±52.56 |
|  | *104y Gal4/+* | 26 | 572.11±47.24 |
|  | *104y Gal4/+; UAS-TfAP2^RNAi^(v101552)/+* | 28 | 569.50±45.37 |
|  | *R23E10 Gal4/+* | 32 | 583.44±36.33 |
|  | *R23E10 Gal4/+; UAS-TfAP2^RNAi^(v101552)/+* | 30 | 564.14±44.66 |
|  | *sNPF Gal4 (#49852)/+* | 32 | 448.67±37.94 |
|  | *sNPF Gal4 (#49852)/+; UAS-TfAP2^RNAi^(v101552)/+* | 29 | 457.73±42.15 |
|  | *TfAP2 Gal4 (#48892)/+* | 16 | 553.38±38.25 |
|  | *TfAP2 Gal4 (#48892)/+; UAS-TfAP2^RNAi^(v101552)/+* | 21 | 546.52±36.86 |
|  | *UAS-TfAP2^RNAi^(v101552)/+*  *Tdc1Gal4/+*  *Tdc1Gal4/ UAS-TfAP2^RNAi^(v101552)* | 21  15  28 | 571.71±34.07  424.46±41.65  447.23±37.95 |
| 2 | *UAS-TfAP2^RNAi^(v101552)/+* | 17 | 527.72±66.90 |
|  | *elavGal4;; tubGal80^ts^* | 20 | 465.07±57.75 |
|  | *elavGal4;; tubGal80^ts^/UAS-TfAP2^RNAi^(v101552)* | 26 | 439.52±53.11 * |
|  | *elavGal4;; tubGal80^ts^*  *UAS-TfAP2/+*  *elavGal4;; tubGal80^ts^ /UAS-TfAP2* | 15  10  20 | 524.84±39.32  500.88±46.33  505.46±34.10 |
| 3 | *UAS-TfAP2^RNAi^ (v101552)/+* | 30 | 321.20±23.93 |
|  | *elav Gal4;; UASdicer2* | 30 | 306.82±38.23 |
|  | *elav Gal4/+;;UASdicer2/UAS-TfAP2^RNAi^(v101552)* | 30 | 134.72±21.24 *** |
|  | *UAS-TfAP2^RNAi^ (v41130)/+* | 30 | 388.53±25.48 |
|  | *elav Gal4;; UASdicer2* | 30 | 477.36±36.66 |
|  | *elav Gal4/+;; UASdicer2/UAS-TfAP2^RNAi^(v41130)* | 30 | 299.33±29.20 * |
|  | *UAS-TfAP2/+* | 30 | 228.76±19.20 |
|  | *elav Gal4/+* | 30 | 410.06±25.96 |
|  | *elav Gal4/+;;UAS-TfAP2/+* | 30 | 253.86±28.06 |

**Supplemental Table S2. Sleep phenotype quantification for TfAP-2 RNAi and overexpression. “**1” denotes that experiments were performed at 25 ºC. “2” denotes that experiments were performed at 29 ºC. “3” denotes that experiments were performed using video recording method. Statistics was calculated with one-way ANOVA and Dunnet’s posthoc tests, * p≤0.05. *** p≤0.001
